# Challenges and Opportunities in Machine Learning for Light‐Emitting Polymers

**DOI:** 10.1002/marc.202500850

**Published:** 2026-01-06

**Authors:** Tian Tian, Yinyin Bao

**Affiliations:** ^1^ Department of Chemical and Materials Engineering University of Alberta Edmonton AB Canada; ^2^ Alberta Machine Intelligence Institute (Amii) Edmonton AB Canada; ^3^ Department of Chemistry University of Helsinki Helsinki Finland; ^4^ Department of Chemistry and Applied Biosciences ETH Zurich Zurich Switzerland

**Keywords:** data‐driven methods, light‐emitting polymers, machine learning, OLED, thermally activated delayed fluorescence

## Abstract

Light‐emitting polymers (LEPs) combine the luminescent properties of organic emitters with the structural versatility of polymers, supporting applications in solid‐state display, chemical sensing, and bioimaging, owning to the efficient tuning of their performance across multiple scales, from monomer units and chain sequence to solid‐state packing and solution processing. Recent strategies have expanded emission color space, improved quantum yields, and simplified design rules, evolving from traditional π‐conjugated systems to mechanisms driven by aggregation and charge transfer. Yet this multiscale flexibility also creates a vast and complex design space, where the interplay of monomer choice, polymer architecture, and processing methods makes it impossible to exhaustively map their structure–property relationships by empirical means. In this perspective, we review the development of recent design strategies in LEPs, highlighting the key experimental challenges they reveal, and discuss how data‐driven approaches, particularly machine learning, can help navigate this complexity and accelerate the discovery and optimization of next‐generation LEPs.

## Graphical abstract

The performance of light‐emitting polymers emerges from coupled effects of chemical diversity, morphology, and exciton dynamics across multiple length scales. This Perspective reviews recent design strategies and experimental challenges, and discusses how machine learning can unify descriptors, data, and modeling approaches to efficiently navigate complex design spaces and enable accelerated development of advanced light‐emitting polymers.

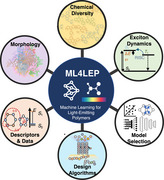

## 1 Overview of Light‐Emitting Polymers (LEPs)

**FIGURE 1:**
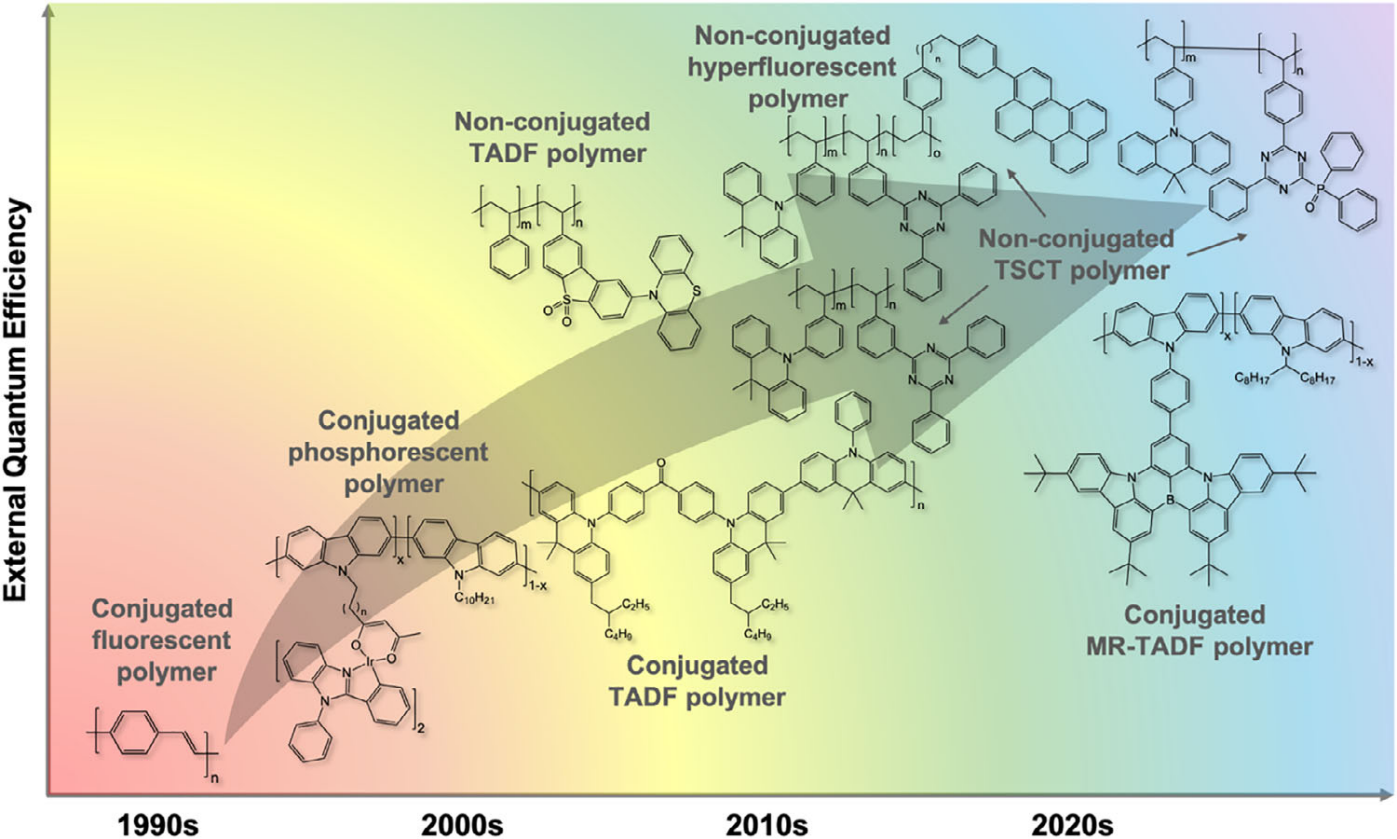
The development of LEPs along with time. Interestingly, the non‐conjugated polymers seem to outperform conjugated polymers for external quantum efficiency (EQE) of OLEDs.

Achieving controllable and highly efficient conversion of electronic excitations into photons is a central challenge across modern materials research, forming the foundation technologies from solid‐state optoelectronics such as light‐emitting diodes (LEDs) [1] to bioimaging [2], diagnostics [3], and fluorescence sensing [4, 5]. Although photon emission can, in principle, be realized in a wide range of inorganic and organic systems, practical applications require materials that combine high efficiency with structural tunability, reliable synthetic control, and compatibility with scalable processing. LEPs address these demands across multiple levels: in addition to the solution‐processing ability, their monomer electronic structures can be tuned using chemical design strategies established in small‐molecule emitters; advances in controlled polymerization techniques [6, 7] enable regulation of polymer chain length and monomer sequence; their emission properties can be further influenced by mesoscopic aggregation and packing effects [8, 9, 10]. A representative and widely applied class of LEPs are π‐conjugated polymers [11] (Figure 1) used in organic light‐emitting diodes (OLEDs) containing aromatic or heteroaromatic backbones to provide extended π‐electron delocalization. These materials are considered the first generation of organic electroluminescent (EL) materials, which combine the dual advantages of efficient synthesis via cross‐coupling polycondensation [12] and convenient solution‐based fabrication [13].

One central challenge of conventional, purely fluorescent π‐conjugated LEPs is that their electron–photon conversion efficiency is fundamentally constrained by spin statistics: only 25% of the excitons are singlets that decay into emissive photons, while the remaining 75% triplets that are non‐emissive in the absence of heavy‐atom effects due to a large singlet–triplet energy gap (Δ*E*_ST_) [14]. Extensive studies of molecular fluorescence pathways have motivated material design strategies to circumvent this singlet–triplet barrier in internal quantum efficiency [15]. A widely adopted approach without incorporating noble metals that are typically used in phosphorescent polymers (Figure 1) [16, 17], is thermally activated delayed fluorescence (TADF) [18, 19], which promotes reverse intersystem crossing (RISC) from triplet state (T_1_) back to excited singlet state (S_1_), enabling theoretical 100% conversion efficiency [20]. Achieving a practical RISC kinetics (*k*_RISC_) requires a Δ*E*_ST_ <0.5 eV [21] for the thermal energy at room temperature to drive the triplet‐singlet transition. This is usually achieved by molecular design, where the highest occupied (HOMO) and lowest unoccupied molecular orbitals (LUMO) are spatially separated [22]. We further note that controlling orbital alignment in molecular design is not the sole determinant of TADF performance. In practical OLED devices, the efficiency roll‐off [23], which is the drastic decrease in luminescent efficiency at higher current densities, also presents a major challenge. Recent studies [24] highlight that the figure of merit governing roll‐off cannot be captured by energetic descriptors alone, but instead arises from the coupled influence of the singlet–triplet equilibrium constant (*K*_eq_) and the radiative decay rate from *S*_1_ ($k_{r}^{S}$). These insights reveal a more complex, multivariate landscape of the structure–property relationships relevant to TADF OLEDs that extend beyond classical donor–acceptor orbital engineering, but may still be decoupled into a combination of single property descriptors.

Similar to the twisted donor–acceptor (D–A) geometries used in small molecule TADF emitters [25], LEPs that make use of the TADF strategy can be realized via a general strategy, through non‐conjugated backbones or side‐chain architectures that bring the donor and acceptor groups into proximity while preventing strong orbital overlap (Figure 1) [26]. These through‐space charge transfer (TSCT) arrangements [26, 27, 28, 29] are particularly well suited to polymers: donor and acceptor moieties positioned as pendant groups or in non‐conjugated copolymers can form charge‐transfer states with reduced Δ*E*_ST_, producing efficient TADF emission in the solid state, without sacrificing the photoluminescence quantum yield (PLQY) [9]. Alternatively, TADF chromophores can be embedded directly into the polymer backbone together with conventional monomers [30], or host‐like units, yielding “self‐hosted” polymers [31] in which intrachain energy transfer channels excitations to the emissive centers.

A further development combines the TADF mechanism with conventional fluorescence through Förster resonance energy transfer (FRET) [32]. In these so‐called hyperfluorescent polymers (Figure 1) [33], the TADF component acts as a sensitizer that captures both singlet and triplet excitons, while the fluorescent component serves as the final radiative center with a high emission rate and narrow spectral bandwidth. This hybrid design combines near‐unity exciton harvesting with bright and spectrally pure emission, and can be implemented in polymers by covalently linking or copolymerizing TADF donors and fluorescent acceptors within the same macromolecular framework.

In parallel with the rapid development of TADF LEPs, aggregation‐induced emission (AIE)‐active polymers [10, 34] have received comparable attention in recent years. This class of polymers addresses another critical challenge in light‐emitting materials: the loss of quantum efficiency when moving from solution to the solid state, where aggregation‐caused quenching (ACQ) typically suppresses emission. In polymers, spatial confinement of AIE‐active moieties along the backbone or as side groups effectively restricts intramolecular motion and mitigates *π*–*π* stacking, converting aggregation into a pathway that enhances rather than quenches emission [35, 36].

Molecular design has progressed well beyond the archetypal tetraphenylethylene (TPE) motif to include a wide variety of donor–acceptor scaffolds, and more recently the generalized class of clusteroluminescent [37, 38] AIE molecules, where through‐space interactions in non‐conjugated systems give rise to efficient solid‐state emission. Within these architectures, through‐bond charge transfer (TBCT) [39, 40, 41], TSCT, and FRET strategies are frequently integrated to tune both emission wavelength and quantum yield in AIE‐active polymers. These developments highlight that, across AIE‐active polymers, charge‐transfer and energy‐transfer processes remain the central levers for manipulating emission color and efficiency.

While the examples of incorporating TADF and AIE feature based on TSCT, FRET, or other mechanisms into LEPs have demonstrated their potential in optoelectronics and biomedicine, translating these design strategies into polymer systems is still relatively new and less mature than in small molecules. Polymer architectures offer unique freedom to combine donor, acceptor, and linker motifs beyond the limits of discrete molecules, but this flexibility also introduces additional complexity in predicting emission properties. In this perspective, we will outline several challenges faced when optimizing for LEP, and how machine learning may be used for tackling these issues, reflecting the viewpoints from both an experimental chemist and a machine learning (ML) scientist for cheminformatics.

### 1.1 Challenges for LEP Design

When considering the emission properties of charge transfer‐based molecules, HOMO and LUMO levels of the electron donors and acceptors have been standard quantification parameters for evaluating the energy bandgaps (*E*_g_) [42]. Adjusting the electron density of the donor and acceptor groups is a common way to tune the HOMO and LUMO levels, resulting in varied energy bandgaps and emission wavelengths [29]. For TADF molecules, maximizing the *k*_RISC_ is crucial to enhance delayed fluorescence. As aforementioned, a small energy gap between S_1_ and T_1_ (ΔE_ST_) is the key as $k_{\mathrm{RISC}}\;\propto e^{-\frac{\Delta E_{\mathrm{ST}}}{RT}}$. A library of donor and acceptor units that can meet these requirements have been established in literature [43], including conventional TADF scaffolds and also the emerging multi‐resonance TADF (MR‐TADF) [19, 44, 45] design using fused heterocyclic aromatic rings. In parallel, AIE fluorophores have been well generated following the restriction of the intramolecular rotation and vibration principle. The charge transfer effect can be conveniently applied to the existing AIE scaffolds to manipulate the emission properties [46], via a similar donor‐acceptor game playing.

Building on TADF or AIE molecules, the polymer design strategy enables a more flexible combination of donors and acceptors, beyond the direct π‐conjugation or close covalent linkage. FRET activity can be further introduced when the absorption and emission can be matched within the polymer backbone/side groups. Certainly, we have a large pool of TADF [42] or AIE [46] active units consisting of donors, acceptors, and linkers, however, to select desirable combinations within polymer systems for achieving target applications is not straightforward, due to the numerous options. By empirical methods, researchers can explore certain combinations, but this trial‐and‐error process is often time‐consuming and may not always yield optimal results. The large number of potential configurations of donor, acceptor, and linker units creates a complex landscape where predicting the performance of any given polymer system becomes increasingly difficult. For example, the *π*‐conjugated polymers and non‐conjugated polymers can show completely different TADF properties. Recent combinatorial study on TADF polymer also revealed that the polymer topology and steric effect largely dominate the photoluminescent behavior [47]. In addition, each molecular unit can interact differently depending on its position within the polymer chain, the presence of neighboring units, and the overall architecture of the polymer. For example, the unexpected polymer end group generation has created a significant charge transfer effect, affecting the final AIE emission of the polymers, despite the minimal difference of only two atoms [48].

In general, while the polymerization strategies leveraging TADF or AIE molecules offer exciting possibilities for creating advanced polymer systems, the optimization process is fraught with challenges. It requires a delicate balance of empirical exploration, theoretical modeling, and advanced synthesis techniques to navigate the vast combinatorial space and achieve the desired material properties for specific applications such as OLEDs. When additional functions like stretchability [49], chirality [50], degradability [51] and stimuli‐responsiveness [52] are needed, the polymer system becomes even more complex. Such complexity, rather than being a limitation, highlights the need for robust, data‐driven methodologies that can scale beyond expert intuition. ML emerges as a natural complement to the experimental design and physical modeling presented above, which has already become essential tool across many areas of materials research. Here we take the position that ML is not only useful for accelerating the development of LEPs, but that the combinatorial and multiscale complexity of LEPs in turn provides an ideal testbed for evaluating how well ML methods can capture structure–property relationships in high‐dimensional materials design.

## 2 Data‐Driven Machine Learning for LEP Design

From the earliest days of polymer science, much of our understanding has centered on formulating governing laws that link molecular characteristics to measurable quantities, such as the Flory–Fox equation [48] that relates polymer molecular weight to the glass‐transition temperature (*T*_g_). In the context of LEP design, the central question is similarly to determine how the polymer's multiscale characteristics, including monomer structure, composition, topology, and chain length, affect the optical properties in solution and aggregate states. Although closed‐form relations may still be attainable through careful multiscale modeling of specific polymer systems (e.g., via tight‐binding / exciton‐coupling treatments) [53], recent efforts in polymer discovery has increasingly turned toward data‐driven ML approaches. A key distinction from classical, law‐based modeling is that ML requires minimal prior assumptions, instead learning structure–property relationships directly from existing experimental or simulation data. At its core, ML for LEP design aims to establish a mapping f:x→y, where x represents a data point in chemical / materials space, and *y* corresponds to one or more targets in the functional space. In supervised learning, *y* consists of explicitly measured or computed properties (such as emission wavelength, quantum yield, or singlet–triplet gap). Unsupervised learning, by contrast, maps inputs *x* from high‐dimensional chemical space to coordinates *y* in a lower‐dimensional latent space, revealing intrinsic relationships or clusters among polymers without predefined property labels.

**FIGURE 2:**
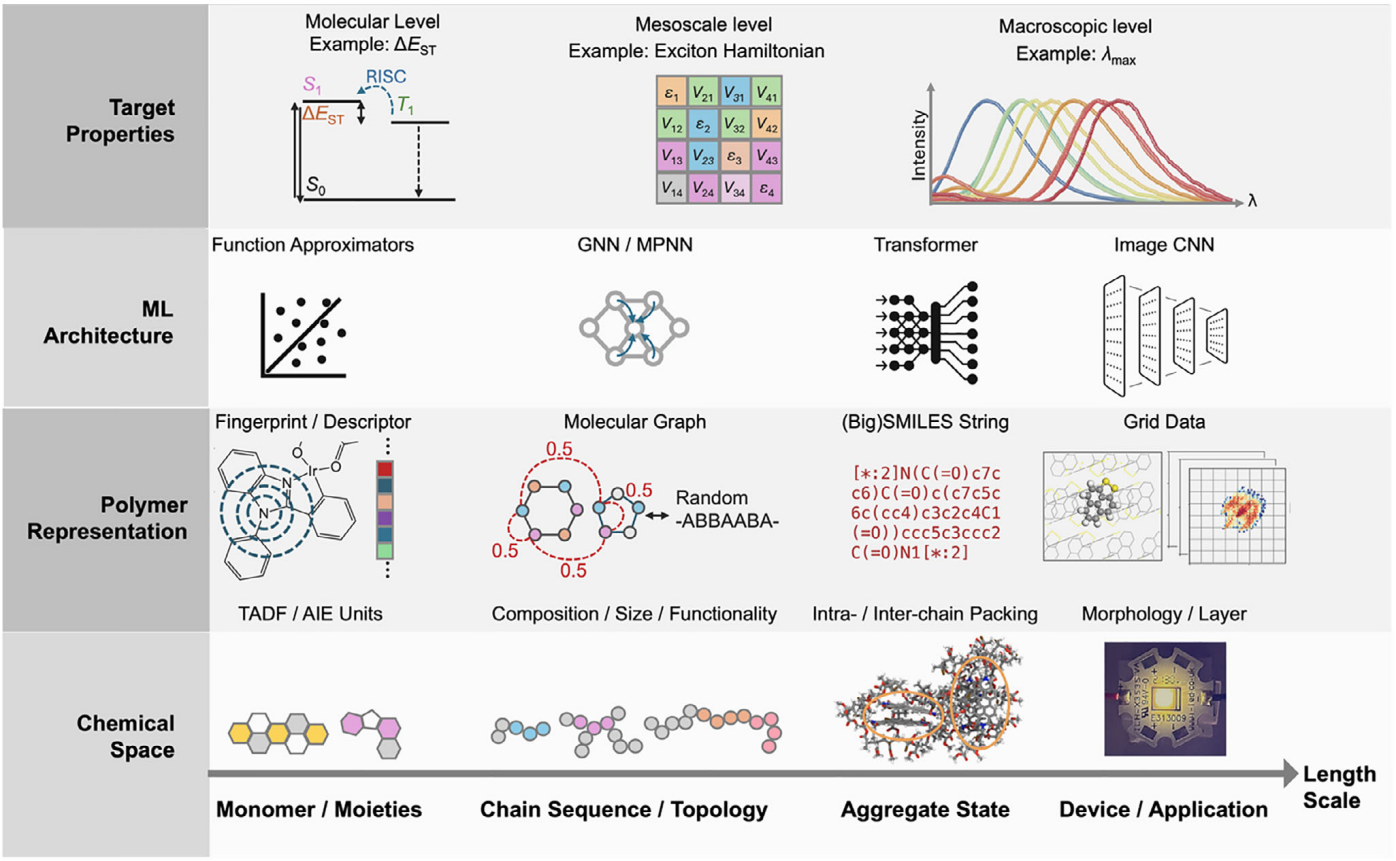
Overview of the representation, model architecture, and choice of target properties when applying machine learning methods to LEPs. Part of the Figure is reproduced with permission from Ref [48] (open access American Association for the Advancement of Science), Ref [] (copyright Elsevier), Ref [45] (open access Springer Nature), and Ref [61] (copyright American Chemical Society).

What makes modern ML applications particularly appealing is their “black‐box” nature: the model architectures act as parameterized function approximators with adjustable weights (conceptually, tunable “knobs”), while the data ultimately define the relationships to be learned.In practice, the structure of these models changes little across domains, while the data provided ultimately defines the relation to be learned. This flexibility has already been demonstrated in polymer informatics [54, 55], where a variety of ML models have shown the ability to generalize across the polymer chemistry space to predict properties including bandgap, glass‐transition temperature, and dielectric constant from curated polymer datasets [56]. Inferencing the x→y relation using the trained models is several orders of magnitude faster than conventional simulations and experiments, making ML powerful surrogate tool that can be embedded at multiple stages of the LEP discovery loop. For a comprehensive overview of machine learning methodologies in chemistry and polymer informatics, the readers are referred to recent reviews covering featurization, model architectures, datasets, and workflow standardization [55, 57, 58, 59]. To our best knowledge, there is no single ML framework that can be directly and universally applied to LEPs, owing to their multiscale structure, stochastic composition, and process‐dependent optical behavior. In this perspective, we therefore focus on several key challenges that arise when adapting ML to LEP systems. Starting from the notation f:x→y, immediate ambiguities appear in how both the inputs and targets are defined. These form the central questions guiding our discussion, which is summarized in Figure 2:

(1) How should molecules and polymers be represented for learning? (*x*)
(2) Which properties or labels are most appropriate for model training? (*y*)
(3) Which classes of ML models best align with the physics and data regimes of LEPs? (*f*)

### 2.1 Molecular Representations for Polymer Learning

Molecular representations define what a machine learning model “sees” when it is given a polymer structure. Although the overall topology of a polymer can be described by its repeat units and connectivity, encoding the entire macromolecule as an input is often impractical. In polymer informatics, a few strategies are often used to implement ML descriptors at different length scales. When the polymer composition is statistical rather than sequence‐defined, monomer‐based descriptors are often sufficient to capture the overall optical property in a donor‐acceptor polymer [62, 63]. A popular choice of creating monomer descriptors is to start from fragment‐based scalar descriptors or vector fingerprints [64], adapted from small‐molecule drug discovery. A wide range of fingerprinting methods based on 2D atom connectivity are often used in ML studies of organoelectronic molecules [65, 66, 67, 68] which encode chemically meaningful functional entities as scalars or vectors. We note that many of the successful applications of monomer based 2D fingerprints work when the dominant electronic and optical properties have a strong correlation with the overall polymer property, including using monomer features for predicting frequency‐dependent dielectric function (*ε*) of homopolymers [69], as well as glass‐transition temperature and electronic bandgap of A‐B alternating copolymers [70]. The same principle extends to LEP discovery. In our recent work [60], we combined monomer‐level chemical fingerprints with experimentally derived features to train small‐scale ML models capable of predicting key photophysical observables, including emission peak wavelength ($\lambda_{\max}$), PLQY, and CIE color coordinates (CIE_xy_). These models allow screening a library of electron‐donating monomer units within the TSCT polymer framework, facilitating the design and synthesis of TSCT polymers with continuously tunable solid‐state photoluminescence across the visible spectrum.

A caveat with monomer‐level fingerprints is that their predictive accuracy often depends strongly on the size and diversity of the dataset. In large polymer informatics studies for dielectric and energy storage applications [70, 71, 72] with datasets on the order of 10^4^–10^5^ entries, the models can typically afford chemical feature space with 10^2^–10^3^ dimensions to capture nonlinear correlations. By contrast, in smaller TSCT polymer datasets [60] with fewer than 10^2^ experimental points, model robustness was achieved only after reducing the descriptor space to around 10^1^ optimized features. A few strategies may be used to boost the performance of descriptor‐based ML models. Combining multiple sets of vector chemical fingerprints (e.g., MACCS, FP2, ECFP) has been shown to increase the model accuracy when screening additives for perovskite LEDs [73]. Many studies shown above also augment chemical fingerprints with polymer‐level information, including molecular weight, dispersity, or compositional ratios. In small‐molecule screening studies for TADF [74] and AIE emitters [75], ground‐state energy descriptors (e.g., energy levels, overlap, and π‐component of molecular orbitals) have been shown to serve as highly informative descriptors for excited state transitions. Whether including these ground‐state electronic descriptors can similarly improve LEP models remains an open question but nevertheless a promising direction.

To avoid the tedious stage of feature engineering (which often constitutes the most design‐intensive step in ML workflows for polymers) [76], a complementary strategy is to embed chemical environments directly through graph representations. In these frameworks, atoms (or monomeric units) are treated as nodes and bonding or topology as edges. Compared with fixed chemical fingerprints, molecular graph representations (often referred to as embeddings) provide greater flexibility in defining what information is included for each node (atomic information) and edge (bonding information). This advantage has been demonstrated in the high‐throughput screening of TADF emitters, where graph embeddings allowed a continuous exploration of chemical space [77] compared with forward‐prediction‐only capability based on 2D fingerprints [78]. Another benefit of graph embeddings over 2D fingerprints is the potential to include distance and angle information from 3D coordinates via continuous filter convolution [79] or spherical harmonic expansion [80]. Coupled with deep convolutional or message‐passing neural networks, graph representations have shown remarkable progress across materials research domains to achieve unprecedented accuracy when trained on large, high‐quality datasets [81, 82, 83]. Without a priori assumptions about feature selection, knowledge transfer from large pre‐trained graph‐based models may offer feasible pathways to accelerate the development of polymer ML models.

Extending the idea of monomer fingerprints, a straightforward approach is to represent the monomer units using molecular graphs, which has been shown to perform decently for homopolymers [84] or alternating A‐B copolymers [85], and often outperforms commonly used ECFP fingerprints [84]. Note in the case of copolymers, the contributions of molecular graphs from individual units are often treated via pooling operations to construct an embedding used for study the polymer properties in a later stage [85]. A more elegant solution to overcome the limitation of monomer graphs was proposed by Aldeghi and Coley [86] by adding stochastic edges (edge weights ranging from 0 to 1) between monomer graphs to represent monomer‐monomer interactions in real polymer topology, analogous to the handling of periodic boundary conditions used in crystal graph neural networks [87]. This molecular ensemble approach can capture diverse monomer compositions, chain architectures, and stoichiometries, achieving over an order‐of‐magnitude improvement in the prediction accuracy of electron affinity and ionization potential compared with monomer‐based fingerprint models. It is worth noting that the definition of edge connections in such periodic polymer representation is often non‐unique, which can be mitigated through data augmentation strategies to include training data of varying chain length [88] or creating invariant graph representations.

For LEPs, where the order of repeat units plays a decisive role in charge transfer and light‐emitting behavior [48, 89], representations capable of capturing sequence information become more critical than in random copolymer systems. String‐based representations offer the advantage of conciseness and memory efficiency compared with descriptor‐based approaches in such cases. A widely adopted format for small molecules, the SMILES notation [90], can be readily extended to polymers by introducing polymerization‐site, as in the p‐SMILES scheme for linear polymers [91]. A more generalized version, BigSMILES [92], extends this concept by incorporating stochastic repeating unit notations that can model a much wider range of polymers. Recent studies have shown that BigSMILES representations generally outperform p‐SMILES and in predicting a variety of polymer properties [93]. One advantage of string‐based notations is their natural alignment with the natural language processing (NLP) paradigm: when coupled with the attention mechanisms implemented in transformer architectures [94], these ML models are capable of capturing sequence‐context relationships directly from tokenized chemical strings [95]. Another long‐established strategy in polymer modeling is the coarse‐grained (CG) representation [96], which groups atoms into effective interaction “beads” to reduce the degrees of freedom while preserving essential structural and energy information. Unlike string‐based notations such as BigSMILES, CG representations are typically deterministic to each polymer sequence, explicitly encoding the spatial arrangement and connectivity of repeat units. When coupled with coarse‐grained molecular dynamics (CG‐MD), the CG representation can capture mesoscale assembly and morphology evolution orders of magnitude faster than all‐atom simulations, which can be crucial for studying LEPs with TSCT and AIE mechanisms [97]. Conceptually, CG bead networks can also be viewed as graph representations, where each node corresponds to a fragment or monomer unit connected through effective interactions [98]. The main challenge in CG modeling lies in parameterization and partitioning [99], yet once established, such representations serve as a powerful embedding tool to survey the polymer space with sequence‐wise precision [100].

### 2.2 Defining and Selecting Target Polymer Properties

Although the ultimate goal in LEP design is to synthesize polymers with desired device performance, in practice, it is often more effective to define labels *y* at multiple physical scales, where the data quality and interpretability differ. A common split is between the ultimate target properties (e.g., PLQY, $\lambda_{\max}$, CIE_xy_) and intermediate properties (e.g., HOMO‐LUMO gap $E_{g}$, $k_{\mathrm{RISC}}$, $\Delta E_{\mathrm{ST}}$) in ML for excited state studies [101].

Thanks to recent advances in computational chemistry [102], intermediate‐property labels are far more abundant, though the majority of high‐quality data for light‐emitting systems remains at the monomer level. The photoluminescence maximum can be approximated using time‐dependent density functional theory (TD‐DFT) as the vertical absorption energy between the ground S_0_ and first excited singlet S_1_ states. By contrast, calculating the excitation kinetics directly from first principles is more demanding [103], and many studies estimate the TADF rate indirectly via analytical relationships linking the oscillator strength to $\Delta E_{\mathrm{ST}}$, which can be computed without co‐optimization of oscillation strength and energy level split [78]. In our work for TSCT polymers, we extended excited‐state simulations beyond the monomer level, demonstrating that TD‐DFT remains tractable for TSCT oligomers containing up to 8–10 repeating units [48], albeit at considerable cost (>10^3^ CPU hours per oligomer). To mitigate such computational expense, physics‐ or chemistry‐informed ML approaches offer an alternative by treating intermediate quantities in well‐established governing relations as learnable variables. For example, for kinetic processes that follow the Arrhenius rate law $k=Ae^{-\Delta E_{a}/RT}$, the activation energy $\Delta E_{a}$ and pre‐exponential factor A can be treated as independent learnable properties within an ML framework. This approach has been demonstrated in the chemistry‐informed ML prediction of temperature‐dependent ionic conductivity of polymer electrolytes [104]. A similar strategy could prove valuable for TADF LEP design, where rate‐determining parameters like *k*_RISC_ follow similar physical principles. Another approach, as already covered in the molecular representation section, seeks to establish ground‐state descriptors as proxies for predicting excited‐state properties [74, 75], offering much faster computation than TD‐DFT computations. It is important to note, however, that the accuracy of such models depends on the validity of the underlying physical correlations and can be biased by systematic errors in first‐principles calculations, including the known over‐ or underestimation of energy gaps due to choices of exchange–correlation functionals [105].

On the other hand, the ultimate or device‐level properties may be more direct performance quantifications, but remain the most difficult to model due to both the time consumption of experimental measurements and their sensitivity to synthesis and processing. Most established polymer databases such as Khazana [56], PolyInfo [106], and PolyDAT [107] focus on bulk thermophysical or electronic quantities, including *T_g_*, *ε*, and *E*_g_. For LEP discovery where optoelectronic properties are important, high‐throughput and automated synthesis [108, 109] and measurement platforms [110] will help accelerate the closed‐loop discovery of novel LEPs. Additionally, the synthetic feasibility of polymers [111] can be considered either as extra ultimate property labeling or constraints to guide experimental exploration.

An often‐overlooked opportunity in property selection of polymer informatics lies in mesoscale excitonic properties, where the aggregation, emitter orientation, and orbital overlap may strongly influence LEP luminescence [89]. In fact, we believe this represents one of the unique challenges where LEP research can contribute to ML development for multiscale systems. One potential route is to model excitation phenomena using tight‐binding or Frenkel‐type exciton Hamiltonians, as applied in π‐conjugated polymers [53], light‐harvesting complexes [112, 113, 114], and AIE aggregates [115, 116]. In such systems, the excitation energies correspond to the eigenvalues of the exciton Hamiltonian, while its parameters (including site energies, coupling strength, and their derivatives) encode local electronic structure and intermolecular interactions. These quantities can be treated as learnable properties within an ML framework. More promisingly, once the Hamiltonian matrix is determined, solving the eigenvalue problem is generally orders of magnitude faster than full‐scale TD‐DFT simulations while preserving the accuracy [113]. Determining the appropriate Hamiltonian formulation, however, requires careful consideration for systems with variable aggregate sizes or site definitions [117], and the Hamiltonian parameters themselves depend strongly on molecular positions and orientations [112], requiring additional molecular or mesoscale simulations to generate representative configurations. Despite these challenges, these Hamiltonian‐informed ML frameworks offer a promising direction for capturing mesoscale excitation processes in LEPs.

### 2.3 ML Model Architectures for Forward Prediction

Once the representations and target properties are defined, model architecture selection becomes relatively straightforward, thanks to the plethora of open‐source ML frameworks that implement most ML function approximators out of the box. In this section, we will discuss the use of forward ML models for property prediction, that is, given a known polymer presentation (x), the ML model approximates the relation f to predict a target property y=f(x). In practice, differences among models only become meaningful when one or more ML models clearly demonstrate the capability to capture the underlying patterns in the data distinctively from others. For a comprehensive overview of commonly used models, evaluation metrics, and training methodologies in polymer informatics, we refer the reader to several recent reviews [54, 55, 59, 76, 118]. Here, we instead focus on how different representation–property combinations guide the selection of suitable models for LEPs.

In the small‐data regime, which still dominates polymer photophysics studies, the choice of model often hinges on balancing predictive capacity with interpretability, i.e., accurate prediction with moderate model architecture. For descriptor/fingerprint‐based ML applications, the model capability (expressiveness) and computational cost generally increase along the sequence: multivariate linear regression (OLS, PLS) → regularized linear models (LASSO, Ridge) → tree‐based ensembles (random forest, XGBoost) → kernel methods (SVM, KRR) → neural networks and their variants [54]. The interpretability, on the other hand, tends to decrease in roughly the same order. An example can be found in our ML‐assisted TSCT design study [60], the choice of ML algorithms is MLREM [119] (linear regression) and BRANNLP [120] (neural network), respectively, both offering the capability of sparse feature selection. While the BRANNLP, being a nonlinear neural network, provides better regression accuracy, it also requires more descriptors. The linear counterpart MLREM provides a convenient way to interpret the contributions of descriptors (or equivalent chemical environments) to the model outcome via regression coefficients. Such interpretability can be extended to nonlinear models using post‐hoc feature‐attribution methods like the SHapley Additive exPlanations (SHAP), which quantify how individual features influence a model's predictions. Although in principle SHAP can be applied to any ML model, it is most commonly combined with tree‐based methods, including demonstrating the fingerprint‐wise impact on the excitation transitions and oscillation strength [121].

As data volume increases or richer representations are employed, graph‐based representations coupled with graph neural network (GNN) [122] or message‐passing neural networks (MPNN) [86] become particularly valuable. Beyond the fact that graph representations naturally encode chemical bonding and polymer topology through nodes (atoms or repeat units) and edges (bonds or interactions), the real strength of these models lies in the message‐passing mechanism. Starting from an initial embedding $h_{0}$ that describes the basic features of each node, the network iteratively updates these representations by exchanging “messages” between connected nodes. At each message‐passing step i, the new embedding $h_{i+1}$ is obtained as $h_{i+1}=h_{i}+m_{i}$, where the message at such step $m_{i}$ aggregates information from neighboring nodes and edges through a learnable transformation. After several iterations, the final embedding $h_{n}$ encodes not only the local chemical environment but also the longer‐range through‐space interactions that are crucial for charge transfer and emission in LEPs. The exact form of these messages $m_{i}$ and how they are combined across layers is optimized during training, allowing the MPNN to discover interaction patterns directly from data, mitigating the limitation of fixed fingerprints.

In principle, the final embedding $h_{n}\;$ can be coupled with any regression or classification model to predict the target property. This can be achieved by either (i) pool the node embeddings into a polymer‐level embedding vector and connect with a downstream neural network to predict global quantities (e.g., $E_{g}$, $\lambda_{\max}$ and PLQY), or (ii) preserve node‐ or edge‐level properties (e.g., per‐node energy, forces, and partial charges) directly through the multistep message passing process. This flexibility in output design makes GNNs versatile tools for polymer science, equally suited for global performance prediction and local environment analysis. The versatility of GNNs and MPNNs extends beyond direct property prediction: the learned embeddings can be leveraged in various ways, including self‐supervised learning [123], multi‐fidelity learning [124] and transfer learning [125] to combat data scarcity challenges, as well as multi‐task learning where shared embeddings are trained to predict multiple correlated properties simultaneously [88]. Additionally, because the learned embeddings retain their correspondence to atomic or monomeric sites, explainable ML strategies, in particular graph attention [126, 127], provide direct visualization of contribution to the target fluorescence properties by atomic [128, 129] or functional group level [130]. A growing research direction focuses on how to design the read‐out part of a MPNN that translates variable‐sized graph embeddings into features suitable for different target properties, ranging from localized quantities such as HOMO/LUMO energies to global observables like photoluminescence spectra [131]. Integrating graph‐based embeddings with physically informed pooling or hybrid models that incorporate explicit 2D/3D polymer geometries may provide the most interpretable and transferable route toward predictive ML discovery in next‐generation LEPs. Lastly, we we highlight the ability of invariant and equivariant MPNNs to perform node‐level predictions of atomic energies and forces, positions them as universal machine learning potentials (MLPs) for complementing existing polymer force fields (without extensive parameterization), accelerating molecular dynamics and multiscale simulations of LEPs. However, the huge memory and computational demands of applying these models to fully atomistic polymer systems remain a major challenge. CG graph representations combined with GNN architectures may provide a viable path toward scalable simulations [132], though how to balance accuracy, transferability, and computational efficiency in such hybrid frameworks remains an open question.

Another emerging opportunity lies in adapting natural language processing (NLP) models, particularly large transformer architectures, as regression tools for polymer property prediction. Early studies explored simplified string‐based representations (analogous to chemical fingerprints) combined with message‐passing and fully connected neural networks to predict ground‐state properties (HOMO and LUMO energies) of π‐conjugated polymers [62]. With the introduction of SMILES and BigSMILES notations for polymers, token‐based language models can now learn embeddings that capture the underlying chemical syntax, allowing pre‐trained language models to be fine‐tuned for property prediction [133]. Notably, the use of text embeddings is already well established in related polymeric systems such as biopolymers and peptides, where attention‐based transformer architectures have been shown to capture the multiscale sequence–structure–function relationships governing self‐assembly and emergent properties [134]. This parallel suggests similar opportunities for LEPs, in which monomer sequences and electronic motifs might be treated analogously to amino acid chains in proteins. More recent efforts couple these text embeddings directly with large language models (LLMs) [135], enabling multitask predictions of properties such as $T_{g}$ and $E_{g}$ through multi‐head attention mechanisms [95]. However, several open questions remain: how robust these text‐based models are to representation variance, and whether the learned attention weights between repeating units carry genuine chemical meaning. A promising direction is to integrate more structured data formats such as polyDAT [107], which enable semantic learning from curated polymer databases.

The practical lesson in model selection is that, rather than pursuing ever larger models, future progress in LEP informatics will depend on representations and ML architectures that respect the established hierarchy of polymer chemistry, sequence, and processing. Many examples discussed in this section illustrate those effective approaches balance model complexity with data quality and quantity. This philosophy naturally extends into using ML models to design polymers through inverse and closed‐loop strategies.

### 2.4 ML for Inverse and Generative Polymer Design

**FIGURE 3:**
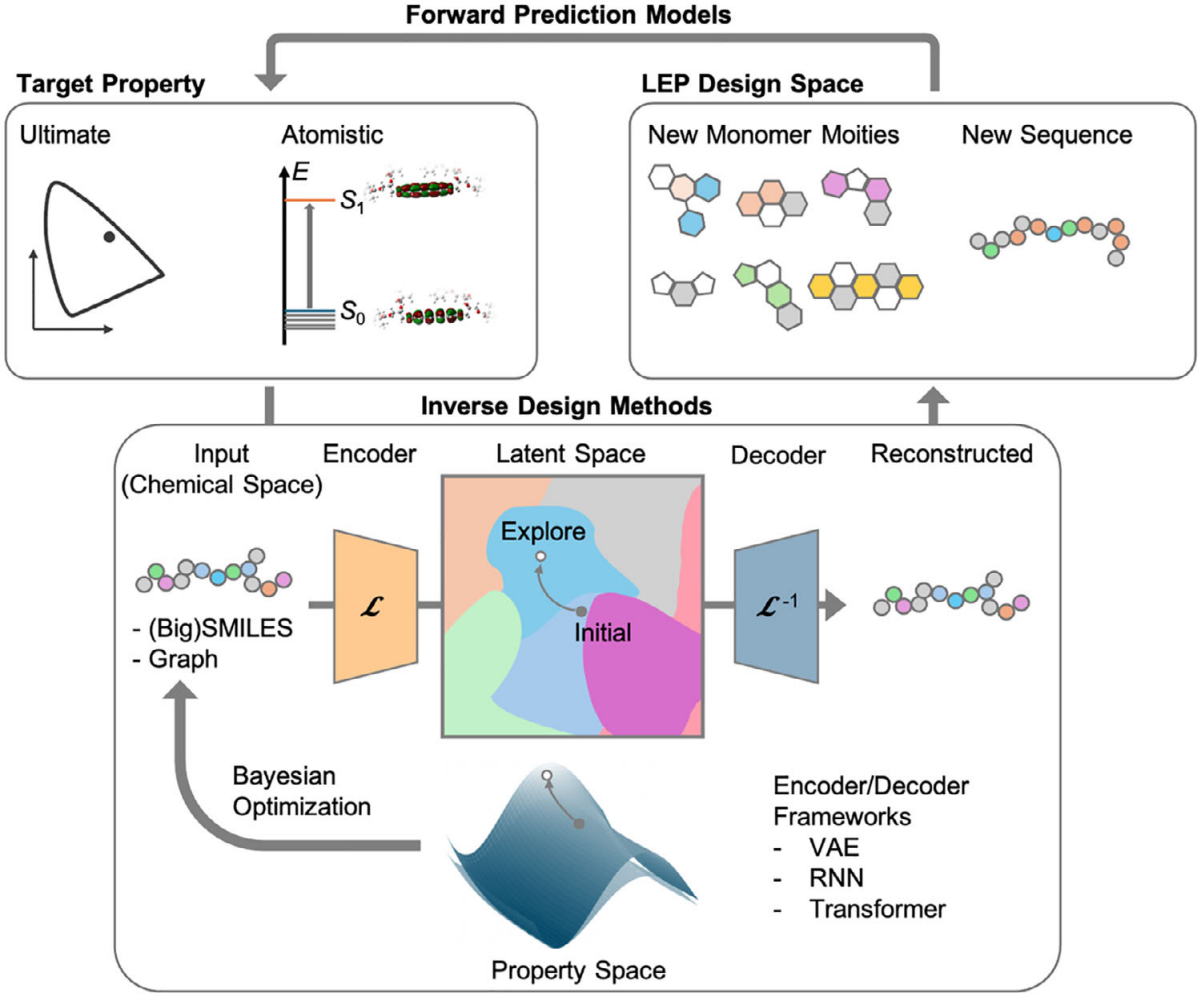
Generalized scheme for ML‐guided inverse design of LEPs. The search for an optimal candidate in the polymer design space, guided by the target property, is performed within the latent space using the forward prediction models as surrogates.

The next milestone in LEP design is to develop algorithms that enable inverse design, that is, given a target property *y*, to identify the optimal polymer structure *x* within the chemical space. For a broader overview of inverse design methodologies in molecular and materials sciences, readers are referred to several seminal reviews in this field [136, 137, 138]. This inverse mapping y→x presents a much greater challenge than the forward‐prediction models (x→y) discussed earlier, reflected by the significantly fewer applications in polymer science than the forward counterparts [138]. Such complexity is particularly pronounced in LEPs, where the design space spans pools of TADF‐ or AIE‐active monomers, possible co‐monomer sequences, chain lengths, and even polymer topologies, making the inverse problem significantly more demanding than analogous tasks in small‐molecule discovery. We generalize such a property‐guided inverse design problem for LEPs in Figure 3.

A simpler version of the inverse design problem may be to select from a bounded design space, for instance, selecting from a known library of TADF or AIE monomers or from a fixed sequence range (such as our monomer optimization for TSCT polymers), when such problem can be formulated as a Bayesian optimization task [139], where the forward‐prediction ML where surrogate ML models iteratively guide the search toward the best‐performing candidates while minimizing the number of ML evaluations. Other evolutionary global optimization algorithms, including genetic algorithm and particle‐swamp optimization, are also frequently for navigate complex polymer composition and sequence spaces [140]. We emphasize that the efficiency of such inverse searches depends critically on the inference speed of the forward‐prediction ML models.

When the design space is open‐ended (i.e. monomers, sequences, or polymer topologies can be designed), the search algorithms can no longer rely on predefined distance metrics between polymer candidates. In such cases, generative models offer a powerful alternative by learning a low‐dimensional latent space that encodes chemical structures in a continuous representation, where distances between points meaningfully reflect similarity in chemical or physical properties. The mapping between input representations and the latent space can be obtained through unsupervised or semi‐supervised training using a variety of polymer representations, ranging from string‐based SMILES/BigSMILES [141] to graph representations. The encoder function is often trained jointly with a target‐property task [77], or using the learned graph embeddings from GNN/MPNN^142^. Sampling within this latent space allows targeted exploration using similar strategies as bounded‐space searches, including Bayesian optimization for property‐guided discovery. Because the latent space is continuous and differentiable, guided traversal from known samples toward optimal regions becomes possible, enabling the discovery of polymers that expand the achievable property frontier [142]. Moreover, when hybrid graph–semantic features are encoded, the encoder–decoder sequence can generate polymers with diverse chain topologies or repeating‐unit distributions [143], or even equivalent string representations from a graph input [144].

Another emerging direction formulates polymer generation as an autoregressive sequence problem, where the model probabilistically predicts the next repeat unit conditioned on existing inputs. Earlier implementations relied on recurrent neural networks (RNNs) or their variants for such sequence generation [91, 145], while more recent transformer‐based architectures such as PolyTAO [146] achieve improved accuracy and chemical consistency by operating directly on tokenized polymer representations. A particularly promising development is the conditional generative approach, where target properties are embedded directly into the input sequence, as demonstrated in retrosynthetic design frameworks for identifying viable molecular building blocks [147]. Extending this idea, recent work in general chemical engineering reformulates design as joint data–property sampling [148], where conditional generation from a learned data–property distribution eliminates the need for an explicit forward model. or LEP design and optimization, insights from these models may be crucial for building robust inverse‐design frameworks, as incorporating practical constraints such as synthetic accessibility, molecular weight distribution, and processability directly into the generative conditions can ensure that the generated polymers are both functionally optimal and experimentally realizable.

## 3 Challenges and Future Directions in ML for LEPs

### 3.1 Capturing Multiscale Physical Phenomena

For LEPs, particularly those governed by TSCT and AIE mechanism, the emissive behavior is dictated not only by molecular design but by solid‐state organization. Our series of studies on TSCT polymers [48, 60, 89] has shown that donor–acceptor proximity, packing orientation, and local confinement strongly influence color purity, emission efficiency, and stability, all of which can be tuned by seemingly subtle variations in polymer backbone rigidity or side‐chain structure. Conventional ground‐ and excited‐state calculations for isolated chains cannot fully capture this multiscale interplay, and ML models trained solely on monomer‐level or single‐chain properties risk missing the dominant physics of emissive aggregates. This complexity underscores the need to curate high‐quality LEP datasets that explicitly reflect aggregation behavior and excitation phenomena at relevant length scales, ideally through high‐throughput molecular dynamics (MD) [149] or coarse‐grained (CG) [96] simulations coupled with quantum‐chemical or TD‐DFT calculations for excited‐state transitions.

ML‐based surrogate models, especially the recent advances of invariant and equivariant GNN/MPNNs as universal MLPs [150, 151] have made it possible to perform dynamics simulations with orders‐of‐magnitude acceleration compared with pure first‐principles approaches. Excited‐state information could, in principle, also be incorporated through transfer learning from models trained on highly accurate small‐molecule datasets [152]. Nevertheless, these ML surrogates should not yet be regarded as direct replacements for quantum‐chemical calculations for LEPs, as their predictions often lack rigorous uncertainty quantification and can easily accumulate during dynamic simulations. Uncertainty‐equipped [153] active learning strategies may offer a promising route forward, which has been demonstrated to accelerate geometric optimization [154, 155] and molecular dynamics tasks [156] while maintaining quantum‐chemistry‐level accuracy. Furthermore, strategies like hybrid simulation methods like QM/MM may be used to accumulate aggregate‐state emission data [157], allow the embedding of emissive fragments within their condensed‐phase environment, while ML‐accelerated CG‐MD force fields extend accessible timescales for aggregation and phase separation. Complementary strategies that decouple certain components in aggregate‐state systems are also helpful in this aspect. In addition to the aforementioned tight‐binding Hamiltonian approaches to parameterize local excitonic couplings, supervised [158] or unsupervised [61] learning methods to extract morphological descriptors from molecular packing can greatly help bridge microscopic and macroscopic observables. In our view, advancing LEP design will require integrated frameworks that combine electronic surrogates, morphological descriptors, and active‐learning‐driven sampling to create physically faithful, multiscale datasets at a feasible computational cost.

### 3.2 Experimental Data Curation and Benchmarking

Despite the rapid growth of polymer informatics and related datasets, curated experimental results for LEPs remain scarce and inconsistent. As discussed in previous sections, most existing databases for polymer property predictions focus on ground‐state or bulk properties, while excited‐state observables are rarely available in a standardized form. Our work on TSCT polymers [60] highlights that constructing meaningful excitation datasets requires careful inclusion of metadata describing synthesis conditions, molecular weight distribution, and aggregation environment. Ideally, these data should also be augmented with solid‐state structural information from recorded morphology and diffraction experiments.

On the experimental side, data generation remains a major bottleneck, constrained by (i) the synthetic effort required to incorporate TADF or AIE functional groups into polymerizable monomers, (ii) the exhaustive screening of compositional libraries, and (iii) the labor‐intensive sequential synthesis and characterization steps. A typical TSCT polymer synthesis cycle can take on the order of several weeks, making large‐scale data collection impractical without automation. Future progress will therefore depend critically on the integration of automated, high‐throughput pipelines [159] capable of controlled polymer synthesis and rapid optical characterization, such as those emerging from flow chemistry and open‐source experimental platforms [160, 161]. ML can play a pivotal role in these autonomous systems by providing fast surrogates for synthesizability and optical‐property prediction, which, when coupled with Bayesian‐inspired experimental planning frameworks [162, 163], can direct experiments toward the most informative candidates, which closely aligns with the inverse‐design strategies discussed earlier. Moreover, experimental datasets need not be built entirely from scratch [164]: multi‐fidelity learning approaches can leverage theoretical or surrogate data (e.g., TD‐DFT or GNN‐based quantum models) as priors to guide which experiments should be prioritized. A few recent examples showcase that transferring knowledge from ML models trained on theoretical data to experimental closed‐loop synthesis can yield interpretable physical insights [165], and incorporating human expertise and decision‐making into this process [78, 165] remains essential for guiding model learning and validating predictions within realistic chemical constraints. Importantly, the goal for ML‐assisted high‐throughput experimental data curation is not merely to expand data volume but to balance simulation and experiment: a single high‐quality TD‐DFT calculation for an oligomer can take as long as an automated photoluminescence measurement. Coordinating these complementary efforts will establish a sustainable data ecosystem for LEP research.

### 3.3 Sequence Awareness and Model Interpretability

Building reliable ML models for LEPs is not only about using these models as black boxes but about balancing predictive capacity with interpretability, which has become a central theme in the materials informatics community [166]. For LEP systems, two considerations are particularly important. The first is whether the ML framework contains sufficient sequence awareness to capture donor–acceptor ordering, end‐group chemistry, and polymer length distribution, all of which are critical for TSCT and AIE emission behavior. The second is whether the predicted properties can be traced back to individual chemical substructures or experimental conditions, allowing the model to provide chemically meaningful explanations rather than statistical correlations.

Apart from the scarcity of well‐sampled, sequence‐resolved LEP datasets, where lessons may be taken from established modeling in biomolecules [167], the core challenge lies in constructing polymer representations that preserve the relevant chemical hierarchy and pairing them with models capable of interpretability. We believe two promising directions could potentially transform the ML study of LEPs, namely, the use of text‐based descriptors such as BigSMILES and its derivatives, and graph‐based representations coupled with message‐passing mechanisms. Both approaches may benefit significantly from incorporating attention mechanisms, where correlations between parts of the sequence or topology are directly learned from the underlying data, paving the way for highlighting the structural contributions to the excitation behaviors of LEPs. Further advances in this field may also come from models with more meticulously designed architectures, including attention‐based frameworks that adaptively assign the range of interactions within each layer [168], the development of physics‐informed readout functions [131], and methods that extract information or physical relations from latent‐space representations [169].

## 4 Outlook and Vision

**FIGURE 4:**
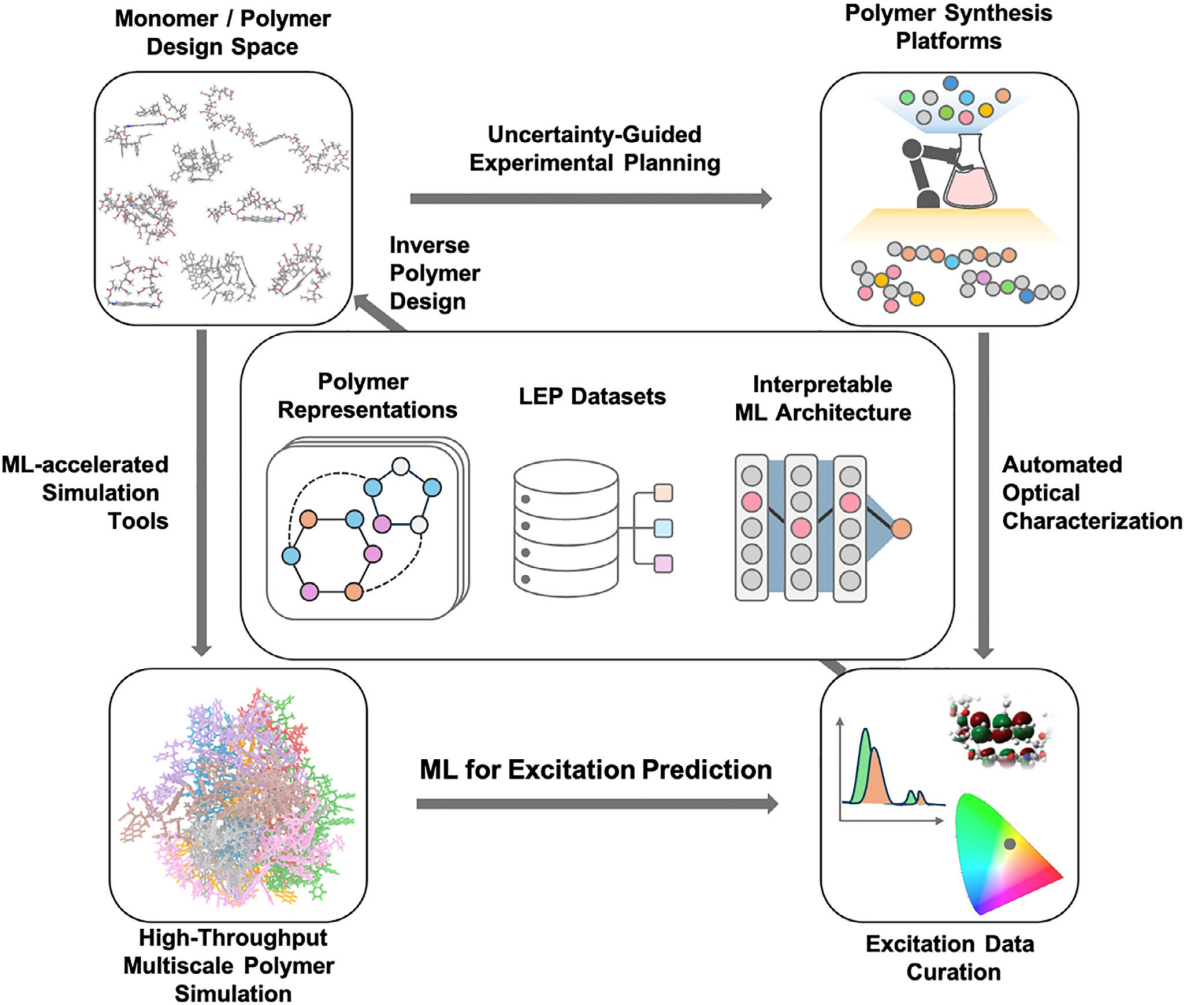
Our vision for incorporating data‐driven ML methods for the closed‐loop design and discovery of LEPs. In this paradigm, ML models connect each component of the loop, while ML‐assisted curation of theoretical and experimental data continuously improves both model development and LEP datasets.

LEPs with new features (e.g., TADF, TSCT, or AIE) are an emerging class of materials with broad potential in energy‐efficient optoelectronics and display technologies. Their immense design space, as the combinatorial product of monomer chemistry, sequence architecture, and aggregation behavior, makes them a compelling platform for advancing multiscale ML methods that connect molecular design with collective emission phenomena. Although current ML applications in LEPs are still at an early stage, rapid progress can be expected as ML acts as an integral component of the discovery loop. Figure 4 summarizes our vision for incorporating ML into the LEP design and discovery loop. Key opportunities include (i) ML‐accelerated multiscale simulations that capture aggregation and excited‐state dynamics, (ii) representations that encode solid‐state behavior without requiring explicit simulation, (iii) transferable models that bridge theoretical and experimental observables, (iv) sequence‐aware inverse design algorithms for optimizing composition and topology, and (v) synthesis‐aware automation that enables self‐driving polymer laboratories. Each of these directions presents distinct challenges, yet together they support our vision of ML not only as a predictive tool but as a central framework for polymeric emitter discovery. As LEP datasets and methodologies continue to mature, the synergy between data‐driven learning and physical modeling will not only accelerate materials discovery but also deepen our understanding of polymer photophysics and multiscale structure–property relationships. These advancements would substantially hasten the development of highly efficient light‐emitting materials for a wide range of optoelectronic devices, especially OLEDs, flexible electronics, and smart displays.

## Funding

University of Alberta; Helsingin Yliopisto; Schweizerischer Nationalfonds zur Förderung der Wissenschaftlichen Forschung 190313; Fondation Claude et Giuliana 1‐005137.

## Conflicts of Interest

The authors declare no conflicts of interest.
